# Integrated Genome-Wide Analysis of MicroRNA Expression Quantitative Trait Loci in Pig Longissimus Dorsi Muscle

**DOI:** 10.3389/fgene.2021.644091

**Published:** 2021-03-30

**Authors:** Kaitlyn R. Daza, Deborah Velez-Irizarry, Sebastian Casiró, Juan P. Steibel, Nancy E. Raney, Ronald O. Bates, Catherine W. Ernst

**Affiliations:** Department of Animal Science, Michigan State University, East Lansing, MI, United States

**Keywords:** microRNA, eQTL, skeletal muscle, pig, GWAS, meat quality

## Abstract

Determining mechanisms regulating complex traits in pigs is essential to improve the production efficiency of this globally important protein source. MicroRNAs (miRNAs) are a class of non-coding RNAs known to post-transcriptionally regulate gene expression affecting numerous phenotypes, including those important to the pig industry. To facilitate a more comprehensive understanding of the regulatory mechanisms controlling growth, carcass composition, and meat quality phenotypes in pigs, we integrated miRNA and gene expression data from *longissimus dorsi* muscle samples with genotypic and phenotypic data from the same animals. We identified 23 miRNA expression Quantitative Trait Loci (miR-eQTL) at the genome-wide level and examined their potential effects on these important production phenotypes through miRNA target prediction, correlation, and colocalization analyses. One miR-eQTL miRNA, miR-874, has target genes that colocalize with phenotypic QTL for 12 production traits across the genome including backfat thickness, dressing percentage, muscle pH at 24 h post-mortem, and cook yield. The results of our study reveal genomic regions underlying variation in miRNA expression and identify miRNAs and genes for future validation of their regulatory effects on traits of economic importance to the global pig industry.

## Introduction

Pork is the meat of choice worldwide, and global meat consumption is projected to continue to increase over the next decade. To fulfill consumer expectations, pork producers will need to continue to improve the quality and consistency of pork products in order to enhance the prosperity of this global industry (USDA, 2020). However, industry focus on increasing carcass leanness has historically neglected meat quality characteristics, increasing the incidence of inferior eating-quality pork and decreasing consumer satisfaction (Barbut et al., 2008; Schwab and Baas, 2008; Ciobanu et al., 2011). Improvements in animal production have been gained through the incorporation of genomic selection techniques (Meuwissen et al., 2016). However, many economically relevant production traits are controlled by complex interactions of gene and protein expression and regulation. While genomic selection has facilitated increased progress on those traits, a better understanding of the genetic architecture controlling phenotypic expression cannot only improve selection in the long term but also enhance management interventions. Improving understanding of the complex regulation of important pork production traits including meat quality, carcass composition, and growth will continue to be a priority for scientists and livestock producers alike.

One genetic mechanism that has been investigated for its role in regulating these economically important traits is the silencing of gene expression *via* microRNAs [miRNA(s) or miR], a class of single-stranded, non-coding small RNA that post-transcriptionally regulate gene expression through sequence complementarity of an approximately 7 nt “seed” sequence with target mRNA sequences. They are known to target genes throughout the genome, influencing a multitude of biological processes. Furthermore, a single miRNA can potentially target hundreds of genes, and multiple miRNAs have the ability to target the same mRNA, acting as “fine tuners” of gene expression and adding rich complexity to the regulation of polygenic traits. Several miRNAs expressed specifically in muscle tissue have been termed the “myomiRs” (miR-1, miR-133a, miR-133b, miR-206, and others), which play critical roles in the proliferation, differentiation, and regeneration of skeletal muscle (for review, see Horak et al., 2016). Previous studies demonstrate miRNA involvement in skeletal muscle development and function in pigs, spanning various developmental time points, physiological states, and breeds (Qin et al., 2013; Siengdee et al., 2013; Cai et al., 2015; Jing et al., 2015; Mai et al., 2016; Xi et al., 2018; Mármol-Sánchez et al., 2020). While many studies have examined effects of miRNAs on diverse phenotypes in pigs, less has been done to elucidate mechanisms regulating the expression of miRNAs themselves, and their effects on downstream phenotypes.

By combining high-density genotypic data with miRNA expression profiles, associations between regions of the genome harboring single nucleotide polymorphisms (SNPs) and the expression of a miRNA can be revealed. These associated genomic regions are termed “miRNA expression Quantitative Trait Loci” (miR-eQTL) and add another layer to our understanding of the regulation of complex phenotypes on the molecular level. Studies identifying miR-eQTL have been reported, but their objectives primarily focus on human biomedical applications, particularly using miRNAs as biomarkers or therapeutics in disease phenotypes (Dong et al., 2010; Gamazon et al., 2013; Huan et al., 2015; Wohlers et al., 2018). These studies reinforce the importance of elucidating the genetic architecture of miRNA expression and its effects on traits of interest to the pig industry.

Understanding both the influence of miRNA regulation on pig production phenotypes and factors affecting the expression of miRNAs themselves will increase our understanding of skeletal muscle tissue at the molecular level, ultimately leading to improvements in pork quality and consistency. Therefore, the purpose of this study was to investigate the regulatory role of miRNAs in growth, carcass composition, and meat quality traits through genome-wide miR-eQTL analyses utilizing small RNA sequencing data from *longissimus dorsi* (LD) muscle samples from an F_2_ Duroc × Pietrain resource population and to integrate gene expression and phenotypic data from the same animals to elucidate regulatory mechanisms controlling these complex traits.

## Materials and Methods

### Data Collection and Sequencing

Animal housing and care protocols were evaluated and approved by the Michigan State University All University Committee on Animal Use and Care (AUF # 09/03-114-00). The 176 animals used in this study were a subset of F_2_ pigs selected from the Duroc × Pietrain Michigan State University Pig Resource Population expressing extremes for loin muscle area or back fat thickness phenotypes (MSUPRP; Edwards et al., 2008a, b; Steibel et al., 2011). These animals were phenotyped for over 60 traits encompassing meat quality, carcass composition, and growth traits. Genotyping was performed by Neogen Corporation—GeneSeek Operations (Lincoln NE) using Illumina PorcineSNP60 BeadChips (Ramos et al., 2009) and was previously reported by our group (Casiró et al., 2017; Velez-Irizarry et al., 2019). Resulting genotypes were filtered for markers with extreme allele frequencies calculated from the entire F_2_ population of 940 animals (*MAF* <0.10), removal of non-informative markers, and markers located on sex chromosomes. A total of 36,292 SNPs were included in subsequent analyses. Genotype data for the animals used in this analysis are publicly available and can be found at https://www.github.com/steibelj/gwaR.

Samples of LD tissue were collected from each animal at slaughter, frozen in liquid nitrogen, and stored at −80°C for later use. Total RNA was isolated from LD samples using the miRNeasy Mini Kit (QIAGEN, California, United States). Samples were prepared for sequencing utilizing the Bioo Scientific NEXTFlex^TM^ Small RNA Sequencing Kit (v2; Bioo Scientific, Austin, TX, United States) and were sequenced on the Illumina HiSeq 2500 platform (Illumina, Inc.; California, United States) in 50 bp, single-end format at the Michigan State University Research Technology Support Facility (East Lansing, Michigan, United States). Sample libraries were sequenced on two flow cells (38 libraries/lane on four lanes of one flow cell; 27 libraries/lane (including 24 new libraries and three repeated libraries) on one lane of a second flow cell), and sequencing of the same library pools was repeated on two additional flow cells to increase sequencing depth. Raw counts of sequence output was merged and compiled into a single file for each sample library prior to analysis. Two libraries failed to produce acceptable sequencing output and were removed from further analysis, yielding 174 files of 50 nt short-read sequences in fastq format (86 male, 88 female). The small RNA sequencing dataset generated for this study can be found in the NCBI Sequence Read Archive under BioProject PRJNA363073.

### Bioinformatics Analysis

After adaptor trimming, read quality filtering, and removal of PCR duplicate reads (Daza et al., 2017), the miRDeep2 software package (v0.0.5; Friedländer et al., 2012) was used with default parameters to align high-quality reads to the *Sus scrofa* reference genome (v11.1; Ensembl), and to quantify *Sus scrofa* mature miRNAs obtained from miRBase^1^ (Release 21; Griffiths-Jones et al., 2008; Friedländer et al., 2012). The average abundance of each mature pig miRNA was adjusted for differences in sequencing depth between libraries by converting the read counts to counts per million (cpm) with the edgeR package (v3.12.1) of R (R Core Team, 2018), incorporating trimmed mean of M (TMM) normalization factors (Robinson and Oshlack, 2010). TMM normalization has been shown to reduce the technical bias of sequencing and control the rate of false-positive associations (Dillies et al., 2013). miRNAs expressed at < 1 cpm in ≥ 44 libraries were removed from the dataset prior to calculation of the normalization factors.

### MicroRNA eQTL Analysis

The 295 mature miRNA expression profiles retained from quality control and expression filtering were normalized using the *voom* function of the *limma* R package (v3.26.9), which accounts for the mean-variance relationship of the miRNA expression profiles across samples (Law et al., 2014). The resulting log-counts per million were treated as response variables in a genomic best linear unbiased prediction (GBLUP)-based genome-wide association (GWA) analysis utilizing the *gwaR* package developed by our group^2^ in the R statistical environment^3^ (R Core Team, 2018). The GBLUP model is described in model (1):

(1) y=X⁢β+a+e,

$$\text{a}∼N\,\left(0,\text{G}\,\sigma_{a}^{2}\right)$$

$$\text{G}=\text{ZZ}{}^{\prime }$$

$$\text{e}∼N\,\left(0,\sigma_{e}^{2}\,\text{diag}\,\left(1/\hat{\text{w}}\right)\right)$$

where ***y*** is the vector of normalized read log-count per million (log-cpm) associated with a mature miRNA; *X* is the incidence matrix relating the observed phenotypes to the coefficients of fixed effects β including the population mean, sex, and “growth group,” which is a factor broken into four classifications corresponding to phenotypic extremes for the selected traits of loin muscle area or 10th rib backfat thickness (Cardoso et al., 2008). Effect ***a*** is the random effect of breeding value, whose variance incorporated the genomic relationship matrix **G**=**ZZ**′, where **Z** is the matrix of standardized SNP marker effects for each animal (VanRaden, 2008; Gualdrón Duarte et al., 2014). The error term **e** included a variance inversely proportional to the precision weights produced by the *voom* function, accounting for the heterogeneous variance between miRNAs with differing expression.

The narrow-sense heritability (*h*^2^) of the miRNA expression profiles was estimated by obtaining the ratio of the additive genetic variance and total phenotypic variance parameters resulting from the GBLUP model as shown in Equation (2):

(2) $$h^{2}=\frac{{\hat{\sigma }}_{a}^{2}}{{\hat{\sigma }}_{a}^{2}+{\hat{\sigma }}_{e}^{2}},$$

A likelihood ratio test (LRT) was utilized to assess the significance of the heritability, comparing the likelihood of model (1) with the null model assuming *h*^2^ = 0. False-discovery rate (FDR) was implemented for multiple test correction (FDR < 0.05; Storey and Tibshirani, 2003). All miRNAs were included in the subsequent GWA analysis, regardless of heritability.

The SNP effects and their variances from model (1) were estimated from a linear transformation of the ***â*** matrix of estimated breeding values, as described by Gualdrón Duarte et al. (2014):

(3) $$\hat{\text{g}}={\text{Z}}^{{}^{\prime }}\,{\text{G}}^{-1}\,\hat{\text{a}},$$

$$\text{var}\,\left(\hat{\text{g}}\right)={\text{Z}}^{\prime }\,{\text{G}}^{-1}\,\text{var}\,\left(\hat{\text{a}}\right)\,{\text{G}}^{-1}\,{\text{Z}}^{{}^{\prime }}$$

Individual SNP effects were standardized by dividing a SNP effect by the square root of its variance, producing the GWA test statistic of the *j*th SNP with a miRNA:

(4) $${\text{t}}_{j}=\frac{\hat{\text{g}}}{\sqrt{\text{var}({\hat{\text{g}}}_{j}})},$$

The *p*-values associated with each significance test were assessed as described in Gualdrón Duarte et al. (2014), and FDR was utilized for multiple test correction (FDR < 0.05).

The miRNA eQTL (miR-eQTL) were then mapped by plotting the genomic position of each miRNA precursor sequence against the associated SNP marker position(s). miR-eQTL were defined as “local” regulators of miRNA expression if the miR-eQTL peak overlapped the genomic position of the miRNA precursor transcript. Peaks not meeting this criterion were defined as “distant” regulators. This definition of local versus distant miR-eQTL is more conservative than that used in traditional mRNA GWA analyses, which generally defines a genomic region surrounding the transcription start site of the associated mRNA (in the range of kilobases) as a “*cis*-acting” eQTL. This conservative definition of local regulators was chosen with the understanding that genomic variation in the region of the miRNA precursor transcript could potentially affect miRNA biogenesis or targeting.

After completing the GBLUP-based GWA analysis using model (1), the significant miR-eQTL peaks were characterized by estimating the proportion of variance accounted for by the peak miR-eQTL SNP (selected based on minimum *p*-value). This was accomplished by conducting a conditional GBLUP-based GWA analysis (Casiró et al., 2017). The association analysis was repeated for each miR-eQTL utilizing Equation (1) and including in the model the genotypes of the most significantly associated peak SNP (determined by *p*-value) as fixed effects. Estimating the proportion of variance explained by the peak miR-eQTL SNP was conducted using Equations (5) and (6), as described in Casiró et al., 2017.

(5) $$\hat{\text{Var}\,\left(q\right)}=b^{2}*\text{var}\,\left(Z_{\text{peak}}\right),$$

where $\hat{\text{Var}\,\left(q\right)}$ is the estimated variance explained by the SNP effect, *b* is the estimated effect of the SNP, and *Z*_*peak*_ is the genotype of the most significant SNP. The proportion of variance explained by the peak SNP is then estimated by dividing the estimated variance of the SNP effect by the total phenotypic variance components as follows:

(6) $$\frac{\hat{\text{Var}\,\left(q\right)}}{{\hat{\sigma }}_{a}^{2}+{\hat{\sigma }}_{e}^{2}+\hat{\text{Var}\,\left(q\right)}}$$

If all other SNP in the region failed to reach significance after fixing the peak SNP for a given miR-eQTL, this indicated the miR-eQTL effect could be explained by a SNP(s) acting in that region. Additionally, if fixing a peak SNP for a peak in one region of the genome caused a SNP association to be eliminated in another genomic region, this indicated that that single SNP was not in linkage disequilibrium with neighboring SNP but was highly correlated with SNP elsewhere in the genome (Casiró et al., 2017). Finally, if significant SNP associations remain after fixing a peak SNP, the associated miRNA expression could be controlled by multiple genomic loci acting independently of one another, with a proportion of variance in miRNA expression being explained by the given genomic region. In summary, this conditional analysis enabled the estimation of the proportion of variance in miRNA abundance explained by the peak miR-eQTL SNP for each miR-eQTL and facilitated the potential discovery of multiple genomic regions significantly associated with variation in miRNA abundance that, due to the effects of linkage disequilibrium, may have originally presented as a single miR-eQTL peak.

### Colocalization of miR-eQTL and pQTL

Previously estimated phenotypic QTL (pQTL; genomic regions significantly associated with variation in a phenotype of interest) from the MSUPRP (Velez-Irizarry et al., 2019) were colocalized with miR-eQTL peaks to identify regions of the genome affecting variation in both miRNA expression and economically important phenotypes. Using the genomic position of miR-eQTL and pQTL peaks, defined as the range between the minimum and maximum significantly associated SNP genomic positions for a given miRNA expression profile or phenotype, multiple types of colocalization events were identified: miR-eQTL peaks completely enveloped by pQTL peaks, pQTL peaks completely enveloped by miR-eQTL peaks, and pQTL peaks overlapping miR-eQTL peaks either upstream or downstream. Once colocalization was identified based on genomic position, the genotypes of the peak SNP of the pQTL were added as a fixed effect in a replication GWA for the miR-eQTL similar to Equation (7), utilizing FDR for multiple test correction (FDR < 0.05). If the previously significant SNP associations of a miR-eQTL were eliminated by adjusting for the peak SNP of the pQTL, it indicated a region of the genome affecting both variation in miRNA expression and phenotypic expression, further supporting a regulatory relationship between the miRNA and phenotype.

### Genomic Colocalization of miR-eQTL-Correlated Target mRNAs and pQTL

To investigate the regulatory mechanisms affecting carcass composition, meat quality, and growth phenotypes in this population, the expression profiles of 15 miRNAs with miR-eQTL were correlated with each miRNA’s target genes’ expression. Prediction of miRNA target genes was conducted with TargetScan using human homologs of pig miRNAs and genes, and the resulting gene lists were filtered to retain genes containing the two most preferentially conserved and effective target site classes (8mer and 7mer-m8; Agarwal et al., 2015). Gene expression profiles were obtained from next-generation sequencing of mRNA from RNA extracted from a subset of the 174 LD samples, yielding 166 individuals with both miRNA and mRNA expression; for detailed methods, see Velez-Irizarry et al. (2019). Expression profiles for miRNA and mRNA datasets (6,628 unique transcripts) were separately adjusted for the same fixed and random effects used in the linear model described in Equation (1). The corrected miRNA and gene expression values output from the GBLUP model were utilized in a two-sided Kendall’s rank correlation analysis in which each miR-eQTL miRNA was correlated with its target mRNAs expressed in this population, and FDR was utilized to correct for multiple tests (FDR < 0.05). Thus, the genomic position of the target mRNAs exhibiting significant negative correlation (the assumed relationship between a miRNA and its targets) were compared with pQTL regions associated with meat quality, carcass composition, and growth phenotypes previously identified in the same population (Gualdrón Duarte et al., 2016; Casiró et al., 2017; Velez-Irizarry et al., 2019). Corrected colocalized target gene expression was also correlated through Pearson’s correlation to the corrected expression of colocalized phenotypes. Covariates included in linear models obtaining corrected phenotypic expression varied by phenotype, following the methods of Velez-Irizarry et al. (2019). An overlap in the genomic positions of a miR-eQTL miRNA’s target mRNAs and a pQTL, in addition to a correlation between the datasets may reveal novel regulatory relationships of miRNAs and mRNAs underlying economically important pig production traits. All scripts utilized in these analyses are publicly available and can be found at https://github.com/perrykai.

## Results

### MicroRNA eQTL Analysis

#### Local and Distant microRNA eQTL

**FIGURE 1:**
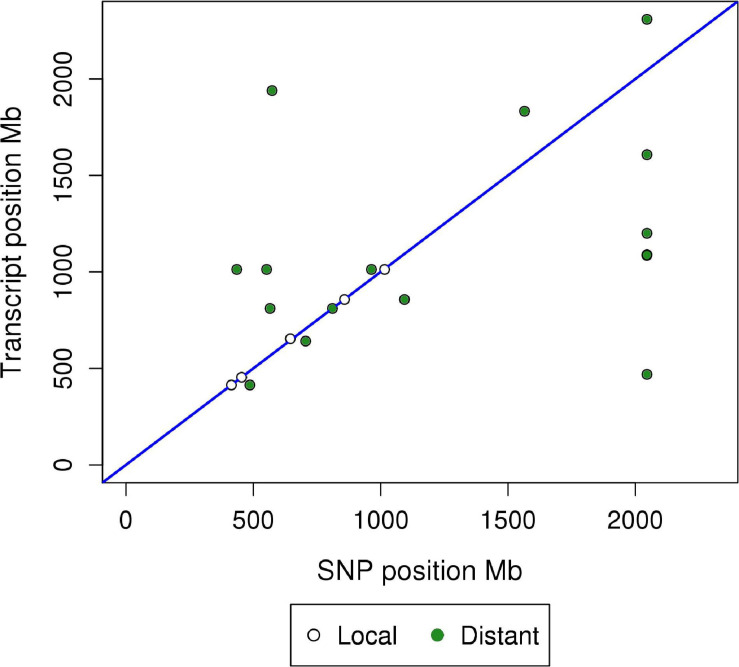
Global plot of genomic position of miRNA transcript (Mb) versus genomic position of SNP (Mb) for miR-eQTL. Significant microRNA eQTL were identified using GBLUP-based GWA models (FDR < 0.05). miR-eQTL were defined as “local” regulators of miRNA expression if the miR-eQTL peak overlapped the genomic position of the miRNA precursor transcript. Peaks not meeting this criterion were defined as “distant” regulators. The *x*-axis denotes the absolute position of the peak miR-eQTL SNP (Mb), while the *y*-axis denotes the absolute position of the miRNA precursor transcript. White points along the blue line represent *local*-acting miR-eQTL, while green points represent *distant*-acting miR-eQTL.

**FIGURE 2:**
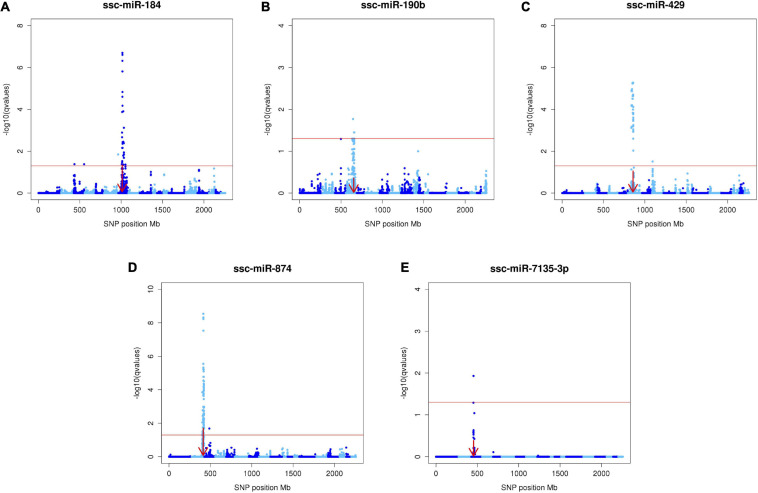
Manhattan plots of the five local-acting miR-eQTL. Manhattan plots depicting the five *local*-acting miR-eQTL identified through GBLUP-based GWA models. For these miR-eQTL [ssc-miR-184 **(A)**, ssc-miR-190b **(B)**, ssc-miR-429 **(C)**, ssc-miR-874 **(D)**, ssc-miR-7135-3p **(E)**], the position of the miR-eQTL peak overlapped the genomic position of the miRNA precursor transcript (denoted by the red arrow). Each blue point represents one miRNA-SNP association, with chromosomes differentiated by shades of blue. The *x*-axis denotes the absolute SNP position (Mb), while the *y*-axis represents the significance of the miRNA-SNP association [-log_1__0_(*q* value)]. The red line designates the threshold of significance, declared at FDR < 0.05.

Results of the miR-eQTL analysis are shown in Table 1. In total, 295 miRNA expression profiles were included as response variables in the GBLUP-based GWA analysis, and results for associating these miRNAs with 36,292 SNPs identified 315 significant miRNA-SNP associations (FDR < 0.05), comprising 23 significant miR-eQTL peaks. These miR-eQTL were associated with 17 unique miRNAs and mapped to 10 different chromosomes. The genomic positions of the miR-eQTL SNPs and their associated miRNA can be found in Supplementary Table 1. The complete map of genomic positions of miRNA transcripts compared with miR-eQTL peak SNPs is shown in Figure 1, with local-acting miR-eQTL in white and located along the blue diagonal line. Five of the miR-eQTL peaks were identified as local regulators, associated with miR-184 (DIAS0000025), miR-190b (ALGA0026452), miR-429 (ALGA0106326), miR-7135-3p (ALGA0124095), and miR-874 (ALGA0016550) as seen in Figures 2A–E, while 16 miR-eQTL were determined to be distant regulators of miRNA expression. None of the significantly associated miR-eQTL SNPs overlapped with mature miRNA sequences. For miR-7135-3p, the significantly associated SNP in closest proximity to the mature miRNA sequence was the peak miR-eQTL SNP (ALGA0124095), lying 16.2 kb away from the mature miRNA sequence. Additionally, while miR-140-5p abundance was associated with two SNPs on chromosome 6 (the same chromosome as the miR-140-5p precursor), the significantly associated SNPs lie approximately 104.5 kb from the miRNA precursor transcript, deeming this association a distant-acting miR-eQTL. This is demonstrated in Figure 1 as the green point lying close to the blue diagonal line.

**TABLE 1:** Summary of miRNA expression quantitative trait loci.

miRNA	miRNA chr.	miRNA *h*^2^ (SE)	Peak SNP ID	SNP chr.	SNP pos. (Mb)	Peak range (Mb)	SNPs in peak	Peak SNP *q-*value	Prop. var. explained peak SNP^a^
miR-874	2	0.30 (0.15)^†^	ALGA0016550^‡^	2	139.74	21.71	115	2.87E-09	0.36
miR-184	7	0.63 (0.17)^†^	DBWU0000430	3	9.46	0.00	1	4.14E-02	0.11
miR-184	7	0.63 (0.17)^†^	ASGA0016793	3	126.51	0.00	1	4.16E-02	0.11
miR-7135-3p	3	6.28E-8 (0.08)	ALGA0124095^‡^	3	28.39	0.70	14	1.17E-02	0.13
miR-874	2	0.30 (0.15)^†^	ALGA0122273	3	61.02	0.00	1	2.06E-02	0.10
miR-9785-5p	NA	0.24 (0.14)	ALGA0121561	3	7.32	27.07	17	3.69E-02	0.14
miR-128	13, 15	0.24 (0.14)	ALGA0023517	4	15.33	0.00	1	4.37E-02	0.17
miR-140-5p	6	0.35 (0.16)^†^	ASGA0017748	4	7.19	0.00	1	1.39E-02	0.15
miR-190b	4	0.50 (0.17)^†^	ALGA0026452^‡^	4	87.03	14.43	4	1.70E-02	0.19
miR-9810-3p	4	0.16 (0.13)	MARC0021620	5	16.31	0.08	2	3.27E-02	0.15
miR-140-5p	6	0.35 (0.16)^†^	ALGA0117081	6	16.97	0.39	2	4.30E-04	0.26
miR-184	7	0.63 (0.17)^†^	M1GA0026172	6	169.93	0.00	1	1.43E-02	0.10
miR-429	6	0.34 (0.16)^†^	ALGA0106326^‡^	6	64.41	23.59	90	5.35E-06	0.28
miR-184	7	0.63 (0.17)^†^	DIAS0000025^‡^	7	51.03	43.23	46	2.00E-07	0.34
miR-429	6	0.34 (0.16)^†^	ALGA0046283	8	7.17	0.00	1	3.10E-02	0.11
miR-6782-3p	NA	0.52 (0.17)^†^	ASGA0094215	10	24.87	9.75	4	1.55E-04	0.26
miR-1306-3p	14	0.03 (0.09)	H3GA0034702	12	52.40	0.00	1	6.13E-03	0.17
let-7d-5p	3	0.11 (0.12)	MARC0093624	15	122.22	0.35	2	1.12E-02	0.18
let-7g	13	0.10 (0.11)	MARC0093624	15	122.22	0.35	2	5.96E-03	0.18
miR-1468	X	0.19 (0.13)^†^	MARC0093624	15	122.22	0.00	1	2.02E-02	0.19
miR-345-3p	7	0.41 (0.16)^†^	H3GA0052416	15	121.81	0.07	2	3.59E-02	0.18
miR-95	8	0.42 (0.16)^†^	MARC0093624	15	122.22	0.41	3	4.65E-02	0.17
miR-9843-3p	8	0.31 (0.15)^†^	MARC0093624	15	122.22	0.41	3	3.23E-03	0.22

*^*a*^Proportion of variance explained by the peak miR-eQTL SNP (minimum p value) when included as a fixed effect in linear models.*

*^†^miRNAs exhibiting significant narrow-sense heritability (FDR < 0.05).*

*^‡^Local-acting miR-eQTL.*

miR-eQTL peaks associated with two miRNAs (miR-6782-3p and miR-9785-5p) could not be classified as local or distant acting; these miRNAs are mapped to unplaced scaffolds in the current genome assembly (v11.1; Ensembl), making this distinction and their inclusion on the global miR-eQTL map in Figure 1 impossible. One SNP, MARC0093624 (SSC15: 122.22 Mb), was associated with five different miRNAs (let-7d-5p, let-7g, miR-1468, miR-95, and miR-9843-3p). This genomic region was found in mRNA eQTL analyses to contain a putative regulatory hotspot (Velez-Irizarry et al., 2019), indicating its potential regulatory role over multiple transcripts. In situations where a miRNA was regulated by both local- and distant-acting miR-eQTL, genomic segments surrounding local-acting miR-eQTL peaks accounted for a larger proportion of variance in its respective miRNA’s expression than distant-acting peak segments.

#### Heritability of miRNA Expression in Pig *longissimus dorsi*

Results of the GBLUP analysis indicated the average *h*^2^ of miRNA expression profiles to be 0.12, while the average *h*^2^ of the 46 miRNAs exhibiting significantly heritable expression after the LRT was 0.34 (FDR < 0.05). The relationship between heritability of a miRNA expression profile and its significance in the LRT [−log_1__0_(*p* value)] is shown in Supplementary Figure 1. The overall trend of this figure (increasing heritability associated with increased significance) indicates that the additive genetic effect is playing a significant role in the expression of these miRNAs in these samples. Heritability of a miRNA expression profile was not used as a filter for inclusion in the GWA analysis; as shown in Table 1, some miRNAs exhibiting non-significant heritability did have miR-eQTL, indicating that a significant association between genomic loci and variation in miRNA expression can exist regardless of the heritability of that miRNA.

### Peak miR-eQTL SNP Conditional Analysis

**FIGURE 3:**
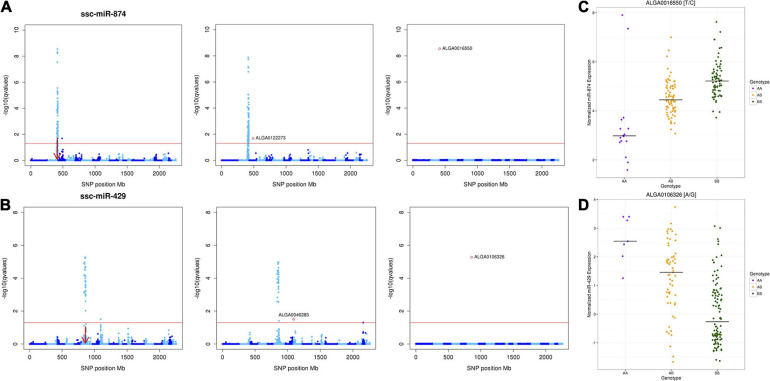
Conditional Analyses of miR-eQTL peak SNPs. Manhattan plots **(A**,**B)** depicting the results of the conditional analyses of miR-874 and miR-429, which included the genotype of the most significantly associated peak SNP (determined by *p*-value) in the GBLUP-based GWA model as a fixed effect for each miR-eQTL. **(C**,**D)** Variations of each miRNA’s expression between segregating genotypes. For each Manhattan plot, the *y*-axis designates the -log_1__0_(*q* value) and the *x*-axis indicates the absolute SNP position (Mb). Chromosomes are differentiated by shades of blue. Significance was declared at FDR < 0.05. **(A)** Conditional analysis of miR-874 miR-eQTL peak SNPs ALGA0122273 (SSC3) and ALGA0016550 (SSC2). **(B)** Conditional analysis of miR-429 miR-eQTL peak SNPs ALGA0046283 (SSC8) and ALGA0106326 (SSC6). Left, Manhattan plot of miR-eQTL peaks. Middle, Manhattan plot of miR-eQTL peaks after conditional analysis of *distant*-acting peak miR-eQTL SNP. Note that the distant-acting peak in each case is eliminated, but the local-acting peak remains. Right, Manhattan plot of miR-eQTL peaks, after conditional analysis of local-acting peak miR-eQTL SNP. **(C)** Variation in miR-874 expression between segregating SNP ALGA0016550 genotypes. The black line in each column represents the median expression of miR-874 for the respective genotype. **(D)** Variation in miR-429 expression between segregating SNP ALGA0106326 genotypes. The black line in each column represents the median expression of miR-429 for the respective genotype.

Results of the conditional analysis (repeating the GWA analysis including the peak SNP for each miR-eQTL as a fixed effect) are shown in Table 2. Strong linkage disequilibrium between the peak SNP and other SNPs comprising the miR-eQTL peak caused the analysis to fail for three miR-eQTL associated with miR-184, miR-7135-3p, and miR-9810-3p (Supplementary Figure 2). For 12 miR-eQTL peaks (representing 12 unique miRNAs), accounting for the peak SNP resulted in complete loss of significant associations. For six of these cases (let-7d-5p, let-7g, miR-128, miR-1306-3p, miR-1468, and miR-345-3p), there was initially only one to two statistically significant SNP association(s) and all were distant-acting. Most intriguing was the local-acting peak associated with miR-874, whose miR-eQTL peaks initially contained 115 significant SNPs (Figure 3A); fixing the peak significant SNP (ALGA0016550) resulted in a complete loss of association for the local- and distant-acting signals. Similarly, for miR-429, accounting for the local-acting peak SNP (ALGA0106326) eliminated the miR-eQTL peak previously consisting of 90 SNPs (FDR < 0.05; Figure 3B). Variation in miRNA expression between segregating peak SNP genotypes for miR-874 and miR-429 are shown in Figures 3C,D, respectively. For the eight remaining miR-eQTL peaks, fixing the peak SNP did not eliminate all SNP associations: these consisted of distant-miR-eQTL associated with miR-140-5p (two peaks), miR-184 (three peaks), miR-429, and miR-874. Additionally, accounting for the miR-6782-3p peak SNP (ASGA0094215; SSC10) revealed significant SNP associations on SSC5 and SSC10 (peaks contain 11 and 6 SNPs, respectively), however, this miRNA lacks annotation data, making the distinction of the resulting eQTL peaks as local- or distant-acting impossible. Interestingly for miR-874, whose expression associated with both a local-acting miR-eQTL on chromosome 2 and a distant-acting SNP on chromosome 3, accounting for the distant-acting miR-eQTL peak SNP in the GWA analysis did not eliminate the local-acting signal but yielded a stronger local signal containing 126 significantly associated SNPs with ALGA0016550 remaining the peak SNP (Figure 3A). A similar result was seen with miR-429, where the local-acting signal remained after conditioning on the distant-acting miR-eQTL peak SNP (Figure 3B).

**TABLE 2:** Summary of miR-eQTL peak SNP conditional analysis results.

miRNA	miRNA chr.	Fixed SNP ID	SNP chr.	SNPs in peak^a^	Peak(s) remaining^b^	SNPs in peak(s) remaining	Remaining peak(s) SNP	Remaining peak(s) SNP chr.
miR-874	2	ALGA0016550^‡^	2	115	0	–	–	–
miR-184	7	DBWU0000430	3	1	2	1, 63	M1GA0026172, ALGA0041952	6, 7
miR-184	7	ASGA0016793	3	1	2	1, 62	M1GA0026172, ALGA0041993	6, 7
miR-7135-3p	3	ALGA0124095^‡^	3	14	NA	–	–	–
miR-874	2	ALGA0122273	3	1	1	126	ASGA0012467	2
miR-9785-5p	NA	ALGA0121561	3	17	0	–	–	–
miR-128	13, 15	ALGA0023517	4	1	0	–	–	–
miR-140-5p	6	ASGA0017748	4	1	1	17	ALGA0117081	6
miR-190b	4	ALGA0026452^‡^	4	4	0	–	–	–
miR-9810-3p	4	MARC0021620	5	2	NA	–	–	–
miR-140-5p	6	ALGA0117081	6	2	1	1	ALGA0022682	4
miR-184	7	M1GA0026172	6	1	2	1, 25	H3GA0011011, ALGA0041952	3, 7
miR-429	6	ALGA0106326^‡^	6	90	0	–	–	–
miR-184	7	DIAS0000025^‡^	7	46	NA	–	–	–
miR-429	6	ALGA0046283	8	1	1	91	H3GA0053081	6
miR-6782-3p	NA	ASGA0094215	10	4	2	11, 6	H3GA0016379, DIAS0000707	5, 10
miR-1306-3p	14	H3GA0034702	12	1	0	–	–	–
let-7d-5p	3	MARC0093624	15	2	0	–	–	–
let-7g	13	MARC0093624	15	2	0	–	–	–
miR-1468	X	MARC0093624	15	1	0	–	–	–
miR-345-3p	7	H3GA0052416	15	2	0	–	–	–
miR-95	8	MARC0093624	15	3	0	–	–	–
miR-9843-3p	8	MARC0093624	15	3	0	–	–	–

*^*a*^Number of significantly associated SNPs in miR-eQTL peak prior to conditional analysis.*

*^*b*^Number of miR-eQTL peaks remaining after conditional analysis of peak miR-eQTL SNP. ^‡^Local-acting miR-eQTL.*

### Colocalization of miR-eQTL and pQTL

Colocalization of miR-eQTL and pQTL based on genomic position of the miR-eQTL and pQTL peak SNPs yielded eight miR-eQTL overlapping 10 pQTL across four chromosomes (Supplementary Table 2). Six distant-acting miR-eQTL for miRNAs let-7d-5p, let-7g, miR-95, miR-345-3p, miR-1468, and miR-9843-3p overlapped in genomic position with a large pQTL peak on SSC15 associated with seven meat quality phenotypes including sensory panel juiciness, sensory panel tenderness, and overall tenderness, Warner-Bratzler shear force (WBS), protein content, pH at 24 h post-mortem, driploss, and cook yield. All but one of these six miR-eQTL peaks were associated with SNP MARC0093624 (122.22 Mb); the miR-eQTL for miR-345-3p was associated with H3GA0052416, located nearby on the same chromosome (121.81 Mb; *r* = −0.89). Two local-acting miR-eQTL peaks for miR-190b (ALGA0026452: SSC4: 87.03 Mb) and miR-184 (DIAS0000025: SSC7: 51.03 Mb) colocalized with pQTL for last lumbar vertebrae backfat thickness (SSC4) and number of ribs (SSC7), respectively. The remaining 15 miR-eQTL peaks did not overlap in genomic position with any pQTL peaks.

For each miR-eQTL colocalizing with a pQTL, the pQTL SNP with the minimum *p*-value was identified and included as a fixed effect in a GBLUP-based GWA conditional analysis to assess the impact of the pQTL peak SNP on the associated miRNA’s expression. Results of this analysis can be found in Supplementary Table 3. For the local-miR-eQTL for miR-184 on SSC7, accounting for the peak pQTL SNP for number of ribs reduced the number of significantly associated SNPs. The number of ribs pQTL SNP ALGA0043983 explained 8.80% of the variance in miR-184 expression, leaving 34 SNP significantly associated with miR-184 (FDR < 0.05; Supplementary Table 3). This means a variant in the locus containing the peak SNP for the number of ribs pQTL is likely also having an effect on the expression of miR-184, further suggesting a regulatory relationship between the miRNA and the phenotype. For the remaining seven colocalized miR-eQTL, including the peak pQTL SNP as a fixed effect in the GBLUP-based GWA eliminated all associations for that miRNA. Interestingly, for miR-190b, which colocalized with last lumbar backfat thickness (SSC4), accounting for SNP ASGA0092651 in the linear model explained 15.67% of the variance in miRNA expression. For the five miR-eQTLs colocalizing with pQTL on SSC15, the peak pQTL SNPs for all seven traits were either H3GA0052416 or MARC0093624 (Supplementary Table 3). As noted above in Table 1, these are the same peak SNPs for the colocalized miR-eQTL, and the two SNPs are highly correlated with one another due to their close proximity in the genome (*r* = −0.89). Conducting conditional analyses on these peak SNPs left no significantly associated SNPs and explained between 13.11 and 21.96% of the variance for a given miRNA (Supplementary Table 3). For the majority of miR-eQTL colocalizing with pQTL peaks, there were only one to four significant SNPs in the original miR-eQTL analysis, so these results are not surprising.

### Genomic Colocalization of miR-eQTL Target mRNAs and pQTL

**FIGURE 4:**
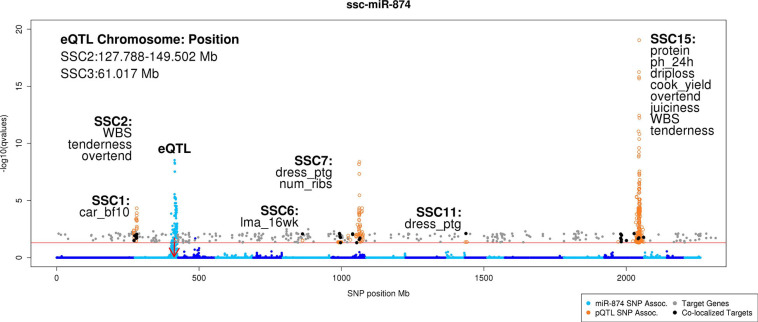
Manhattan plot of genomic colocalization events of miR-874 target genes with pQTL. This Manhattan plot compiles the results of the miR-eQTL, previously identified mRNA eQTL (Velez-Irizarry et al., 2019), previously identified pQTL (Gualdrón Duarte et al., 2016; Casiró et al., 2017; Velez-Irizarry et al., 2019), miR-874 target prediction, and correlation analyses to identify potential novel mechanisms regulating growth, carcass composition, and meat quality traits in pigs. In total, 29 unique target genes colocalized with pQTL across six chromosomes. Nine target genes (*PLEKHB2*, *WDR33*, *NIF3L1*, *IGFBP5*, *UGGT1*, *PRPF40A*, *METTL8*, *PTPRN*, and *KLHL30*) overlap the pQTL peak on SSC15 associated with meat quality traits. The *y*-axis denotes the significance of the association [-log_1__0_(*q* value)], and the *x*-axis indicates the absolute SNP position (Mb). Chromosomes are differentiated by shades of blue. Blue points represent miRNA-SNP associations for miR-874. The red arrow indicates the genomic position of the miR-874 precursor transcript. Orange circles represent the genomic position of pQTL. Gray points represent the genomic position of significantly negatively correlated target genes for miR-874. Black points represent the genomic position of significantly negatively correlated target genes for miR-874 whose genomic position overlaps that of a pQTL peak. Text in the upper left-hand side of plot describes the genomic position of the miR-eQTL peaks (Mb). Text in close proximity to pQTL peaks colocalizing with target genes describe the chromosome on which the pQTL resides and which phenotypes the associated pQTL represents (car_bf10, carcass 10th rib backfat thickness; cook_yield, cook yield; dress_ptg, dressing percentage; driploss, driploss; juiciness, sensory panel juiciness; lma_16wk, loin muscle area at 16 weeks of age; num_ribs, number of ribs; overtend, sensory panel overall tenderness; ph_24h, pH measured at 24 h post-mortem; protein, protein percentage; tenderness, sensory panel tenderness; WBS, Warner Bratzler shear force).

Significant negative correlated target genes were colocalized based on genomic position with pQTL identified in the same population to identify potential novel mechanisms regulating growth, carcass composition, and meat quality traits. Significant miRNA-target correlations ranged from -0.09 to -0.18 (FDR < 0.05). MiR-874 negatively correlated with 29 target genes overlapping with pQTL for 12 phenotypes across six chromosomes (Supplementary Tables 4, 5 and Figure 4). These phenotypes include carcass 10th rib backfat thickness, loin muscle area at 16 weeks old, number of ribs, sensory panel tenderness and overall tenderness, WBS, dressing percentage, protein content, pH at 24 h post-mortem, cook yield, driploss, and juiciness. Correlation analysis between retained target genes and the colocalized phenotype yielded 11 potential targets of interest (Table 3). These genes were negatively correlated with miR-874 corrected expression (FDR < 0.05) in addition to either the corrected gene or corrected miR-874 expression being correlated with the colocalized phenotype (*p* < 0.05). These genes serve as potential subjects to further investigate the role of miRNAs in the regulation of traits important to the swine industry.

**TABLE 3:** Correlations of miR-874, target genes, and colocalized phenotypes.

Gene (chr)	*τ* miR-874: gene^a^	Colocalized phenotype^b^	*R* gene: phenotype^c^	*p-*value^d^	*R* miR-874: phenotype^e^	*p-*value^f^
GPR157 (6)	–0.160	lma_16wk	–0.217	0.005*	0.242	0.002*
CDKN1A (7)	–0.088	dress_ptg	–0.157	0.044*	0.165	0.034*
ETV7 (7)	–0.126	dress_ptg	–0.116	0.135	0.165	0.034*
LEMD2 (7)	–0.148	dress_ptg	–0.126	0.106	0.165	0.034*
NUDT3 (7)	–0.132	dress_ptg	–0.147	0.059	0.165	0.034*
PFDN6 (7)	–0.151	dress_ptg	–0.156	0.045*	0.165	0.034*
ZNF451 (7)	–0.176	dress_ptg	–0.129	0.098	0.165	0.034*
ATP8A2 (11)	–0.174	dress_ptg	–0.115	0.141	0.165	0.034*
IGFBP5 (15)	–0.116	ph_24h	–0.113	0.171	0.169	0.039*
		cook_yield	–0.029	0.716	0.187	0.016*
PLEKHB2 (15)	–0.119	protein	–0.250	0.001*	0.100	0.202
PTPRN (15)	–0.123	ph_24h	–0.231	0.005*	0.169	0.039*
		cook_yield	0.020	0.801	0.187	0.016*
		WBS	0.223	0.004*	–0.009	0.905

*^*a*^Kendall’s rank correlation of miR-874 to target gene (FDR < 0.05).*

*^*b*^Abbreviated phenotype name: lma_16wk, loin muscle area at 16 weeks; dress_ptg, dressing percentage; ph_24h, pH measured at 24 h post-mortem; cook_yield, cook yield; protein, protein percentage; WBS, Warner Bratzler shear force.*

*^*c*^Pearson’s correlation of target gene corrected expression to colocalized corrected phenotype.*

*^*d*^Significance of Pearson’s correlation of target gene-corrected expression to colocalized corrected phenotype.*

*^*e*^Pearson’s correlation of miR-874-corrected expression to colocalized corrected phenotype.*

^*f*^Significance of Pearson’s correlation of miR-874-corrected expression to colocalized corrected phenotype.

*p < 0.05.

## Discussion

This study is the first to report miRNA-eQTL in a livestock species. The regulatory effects of miRNAs in pig skeletal muscle are not yet fully understood. Even less explored is how variation in miRNA expression affects complex traits important to the quality and consistency of pork products and the efficiency of pig production. To elucidate these relationships, we conducted a GBLUP-based GWA analysis of miRNA expression profiles in LD tissue from a F_2_ Duroc × Pietrain pig population. The GBLUP model was used to estimate breeding values for each miRNA expression profile, accounting for population stratification *via* the genomic relationship matrix **G** composed of standardized marker effects for 36,292 SNPs. The GWA analysis was then conducted by estimating SNP effects through a linear transformation of the estimated breeding values and testing their association with a given miRNA expression profile. This resulted in the identification of genomic regions associated with variation in miRNA expression, or conversely, the identification of miRNAs experiencing variation in expression due to additive genetic effects in this population. Results of the miR-eQTL analysis were subsequently integrated with gene expression and phenotypic data from the same animals to elucidate the effects of miRNA regulation on target genes and downstream phenotypes. We identified 23 miR-eQTL peaks (FDR < 0.05) corresponding to 17 unique miRNAs acting both locally and distantly to regulate miRNA expression. Nine of the 17 miR-eQTL miRNAs have previously been linked to biological processes in skeletal muscle and adipose tissues (Shao et al., 2011; Jiang et al., 2013; Yan et al., 2013; Boudoukha et al., 2014; Jeanson-Leh et al., 2014; Shi et al., 2015; Siengdee et al., 2015; Zhang et al., 2015; Dai et al., 2016; Peng et al., 2016; Li et al., 2017; Xie et al., 2018; Zhang et al., 2018; Yu et al., 2019); whereas, others are novel findings for these tissues.

MicroRNAs let-7d and let-7g each exhibited distant*-*acting miR-eQTL overlapping a large pQTL peak for meat quality traits on SSC15. Members of the let-7 family of miRNAs have been shown to be involved in diverse biological processes including glucose metabolism, glycogen synthesis, adipogenesis, and myoblast motility in multiple species. Let-7d was identified as a direct translational repressor of the anti-inflammatory cytokine *IL13* gene, indicating its role in glucose metabolism and glycogen synthesis in human skeletal muscle (Jiang et al., 2013), while another let-7 family member, let-7g, was identified as a candidate regulator of adipogenesis during fetal skeletal muscle development in sheep (Yan et al., 2013). MicroRNA let-7g has also been shown to regulate mouse skeletal muscle myoblast motility (Boudoukha et al., 2014). These reports indicate biological mechanisms through which variation in the expression of these miRNAs could be affecting myogenesis and adipogenesis in pig skeletal muscle, ultimately affecting traits important to pork quality.

Shi et al. (2015) demonstrated that miR-128 inhibited proliferation and promoted differentiation in C2C12 myoblasts through targeted repression of *MSTN*, subsequently promoting expression of the Smad2/3 signaling pathway genes *MYF5*, *MYOG*, and *PAX 3/7*, indicating the critical role of this miRNA in regulating myogenesis. Similarly, Xie et al. (2018) investigated myogenesis-associated miRNAs in C2C12 myoblasts and identified miR-128 as a promoter of differentiation, demonstrating its role in a multi-miRNA network repressing the *MYOD*-inhibiting JNK/MAPK signaling pathway. MicroRNA-128 has also been shown to regulate bovine skeletal muscle satellite cells by targeting *SP1*, as reported by Dai et al. (2016). Upon transfection of cultured fetal hind limb muscle samples with miR-128 mimics, they observed down-regulated SP1 protein levels at late differentiation stages. The inverse relationship also held true when cells were transfected with miR-128 inhibitors, suggesting miR-128 inhibition could promote differentiation in bovine skeletal muscle satellite cells by increasing SP1 protein levels, which promotes *MYOD* expression and the exit of cells from the cell cycle. Additionally, in a study of C2C12 myoblasts, miR-345-3p was predicted to target two genes involved in mitochondrial energy metabolism, suggesting its role in regulating ATP levels during myoblast differentiation (Siengdee et al., 2015). Yu et al. (2019) demonstrated that miR-190b expression decreased in skeletal muscle of rats after burn injury. This suppression of miR-190b resulted in increased abundance of its identified target gene, PH domain, and leucine-rich repeat protein phosphatase 1 (*PHLPP1*). Increased abundance of PHLPP1 protein, the phosphatase of Akt, indirectly enhanced the expression of cell autophagy-related proteins and promoted burn-induced skeletal muscle wasting in this study (Yu et al., 2019). MiR-95 was found to be significantly upregulated in a study profiling miRNAs in serum of golden retriever dogs with muscular dystrophy, which was validated in human patients with Duchenne muscular dystrophy (Jeanson-Leh et al., 2014). This miRNA is located in an intron of the *ABLIM2* gene in dogs and pigs, which encodes a muscle actin-binding protein. Furthermore, after observing increased miR-95 abundance in LD muscle of loss-of-function *MSTN*-mutant Meishan pigs, Li et al. (2017) utilized C2C12 myoblasts to identify its regulatory role in myogenesis. Aminoacyl-tRNA synthase complex-interacting multifunctional protein 2 (*AIMP2*) was found to be a miR-95 target gene, with overexpression of miR-95 inhibiting translation of the protein and promoting myogenic differentiation (Li et al., 2017). While further examination into the function of miRNAs −128, −345-3p, and −95 in pigs would be required, these studies all support possible roles these miRNAs exhibiting eQTL in our study could play in regulating traits of importance to the pig industry.

Skeletal muscle tissue is intercalated with intramuscular adipocytes, so it is logical to also investigate miRNAs affecting adipose tissue when studying skeletal muscle. Multiple miRNAs have been shown to regulate adipogenesis and fat deposition across species including mouse, chicken, pig, and human. MicroRNA 140-5p exhibited increased expression in adipose tissue samples from obese compared with lean mice, and miR-140-5p overexpression in cultured stromal cells promoted adipocyte formation and increased protein levels of adipogenic transcription factors and marker genes. The authors proposed a regulatory model with increased miR-140-5p expression promoting adipocyte differentiation through suppression of its target gene, transforming growth factor β receptor 1 (*TGFBR1*; Zhang et al., 2015). Investigation into the genetic regulation of meat quality traits in chicken breast muscle tissue revealed miR-140-5p as a possible regulator of intramuscular adipogenesis. Overexpression of miR-145-5p in cultured intramuscular preadipocytes resulted in increased lipid accumulation, and luciferase reporter assays confirmed the targeting relationship between miR-145-5p and retinoid X receptor gamma (*RXRG*; Zhang et al., 2018). Comparing sequences of bone morphogenic protein 5 (*BMP5*) between Large White and Meishan pigs revealed a mutation in its 3′ UTR, located in miRNA binding sites for let-7c and miR-184 and associated with fatness traits in a crossbred population of the two breeds. Down-regulation of *BMP5* after transfection of recombinant vectors of let-7c and miR-184 primary sequence into pig fibroblast cells further supported the role of these miRNAs in regulating fat deposition in pigs (Shao et al., 2011). Another study of pig adipogenesis identified miR-429 as a promoter of pre-adipocyte proliferation. Peng et al. (2016) cultured both porcine subcutaneous and intramuscular pre-adipocytes and observed the down-regulation of miR-429 throughout adipogenesis. Overexpression of the miRNA prior to differentiation induction resulted in decreased lipid accumulation in both cell types, and decreased gene and protein expression of adipogenic markers, indicating its role in regulating this process (Peng et al., 2016). MicroRNAs-184 and -429 each exhibited both local- and distant-acting miR-eQTL in this study, while miR-140-5p showed distant-acting regulation. These results indicate the complex regulation of these miRNAs that may, based on the evidence shown here, eventually influence traits related to adipogenesis and fat deposition in pigs.

These studies all indicate ways in which variation in miRNA expression could affect biological processes in skeletal muscle and potentially affect phenotypes of economic importance to the swine industry. With this in mind, in addition to studying the genomic region surrounding the miR-eQTL peaks, we identified the miRNA whose expression was being affected by genomic variants and considered what the functional effects would be on their target gene(s)’ expression and related phenotypes. Target genes were colocalized based on genomic position with pQTL previously identified in the same population of pigs (Gualdrón Duarte et al., 2016; Casiró et al., 2017; Velez-Irizarry et al., 2019), revealing miR-874 as a focus of interest.

The miR-874 primary transcript lies within an intron of the ubiquitously expressed *KLHL3* gene on the reverse strand, which is involved in *innate immune system* and *antigen processing and presentation* pathways and is enriched for *actin binding* and *structural molecule activity* GO terms (Stelzer et al., 2011)^4^. The peak SNP for the local-acting signal explained 36% of the variance in miR-874 expression in conditional analyses, indicating the strong likelihood of an influential variant residing in the region comprising this peak. While the miR-eQTL peak encompasses the miRNA primary transcript, the peak SNP lies 81 kb 5′ of the miRNA primary transcript on the reverse strand. There are no SNPs on the SNP60 BeadChip residing between the positions of the peak SNP and the miRNA primary transcript. Thus, it is likely that the peak miR-eQTL SNP is in high linkage disequilibrium with the true variant controlling variation in miR-874 expression. Further experiments are required to identify this candidate marker and examine the impact of variation on miR-874 expression and downstream phenotypes.

In this study, 29 unique negatively correlated miR-874 target genes colocalized with 12 pQTL across six chromosomes, including carcass backfat thickness measurements and meat quality phenotypes such as tenderness and juiciness. Intriguingly, miR-874 was previously shown to be involved in fat deposition in beef cattle. Guo et al. (2017) profiled miRNAs differentially expressed between Wagyu versus Holstein cattle, two breeds differing in intramuscular lipid content (marbling). Expression of miR-874 was lower in subcutaneous adipose tissue in Wagyu compared with Holstein cattle. Target prediction revealed *RXRA* as a likely miR-874 target, an important transcription factor in the peroxisome proliferator-activated receptor (*PPAR*) signaling pathway involved in lipid and carbohydrate metabolism (Guo et al., 2017). Additionally, Gondret et al. (2016) identified decreased expression of *RXRA* and *PPARG* (its heterodimeric partner) in perirenal and subcutaneous adipose tissues of pigs fed a high-fiber, high-fat diet compared with those fed a low-fiber, low-fat diet, indicating its role in regulating adipogenesis. *RXRA* was corroborated in our study as a miR-874 target and colocalized with a carcass 10th rib backfat pQTL on chromosome 1, supporting the hypothesis that miR-874 is involved in regulation of adipose tissue.

The cyclin-dependent kinase inhibitor 1A (*CDKN1A*) gene colocalized with dressing percentage, a trait that is affected by the overall muscularity and adiposity of an animal. *CDKN1A* functions as a regulator of cell cycle progression and is induced by myostatin in proliferating myoblasts. The upregulation of *CDKN1A* inhibits the cyclin-dependent kinase 2 (CDK2) complex, resulting in hypo-phosphorylation of retinoblastoma protein and the ultimate arrest of myoblasts in the G1 phase of the cell cycle, promoting myoblast differentiation (Thomas et al., 2000). Conversely, in animals lacking functional myostatin, the dysregulation of *CDKN1A* and its associated genes promotes myoblast proliferation, maintaining hyperplasia and resulting in increased muscularity. Thus, variation in *CDKN1A* could contribute to the overall muscularity of an individual (a trait for which the two parental breeds of this population differ), affecting dressing percentage at harvest.

The LEM domain nuclear envelope protein 2 (*LEMD2*) gene is expressed in the inner nuclear membrane of the nuclear envelope, and in our study also colocalized with pQTL for dressing percentage. After depletion of *LEMD2* and its paralog (*LEMD3*) through RNA interference in C2C12 myoblasts, Huber et al. (2009) observed a significant reduction in differentiation as measured by myogenic index (the percentage of nuclei found in MyHC-positive myotubes). After recognizing that *LEMD2* depletion did not affect cell cycle distributions, they utilized western blotting to discover that it instead increased activation of Mitogen-Activated Protein Kinase 3 (MAPK3) a member of the MAPK signaling pathway, thereby suppressing myoblast differentiation (Huber et al., 2009). In our study, the indication that variation in *LEMD2* expression might affect muscle mass would relate to many economically relevant traits in pigs, including dressing percentage.

Insulin-like growth factor-binding protein 5 (*IGFBP5*) colocalized with pH at 24 h post-mortem in our analysis. This gene was identified in a GWAS study of Finnish Yorkshire boars as a candidate gene for 24-h meat pH (Verardo et al., 2017). The authors also discovered an eQTL peak on SSC15 encompassing *IGFBP5*, and subsequent gene-transcription factor network analysis indicated its potential for regulating this trait (Verardo et al., 2017). Offspring of transgenic mice overexpressing *IGFBP5* were shown to exhibit a reduction in skeletal muscle tissue mass of 31% and decreased muscle fiber hypertrophy, predicted to be due to inhibition of IGF-II activity (Salih et al., 2004). Additionally, Ren et al. (2008) knocked down *IGFBP5* expression in C2C12 mouse myoblasts and observed reduced *MYOG* expression and inhibited myotube formation which was not rescuable with exogenous *MYOG* transfection in the absence of IGFBP5. Finally, *IGFBP5* has been shown to be a target of miR-143 in two studies, one assessing its effects on proliferation and differentiation of primary bovine muscle satellite cells (Zhang et al., 2017) and the other investigating muscle regeneration in satellite cells and primary myoblasts from mice and humans (Soriano-Arroquia et al., 2016). The above studies demonstrate the role of *IGFBP5* expression on skeletal muscle development and the potential impacts of miRNA regulation on downstream phenotypes.

Many of the predicted miR-874 target genes identified in our study have not been characterized for their roles in skeletal muscle. Further investigation into the effects of miR-874 and these target genes are needed to fully elucidate its role in pig production traits.

Due to the limited number of recombination events occurring in our F_2_ population, long-range persistence of linkage disequilibrium limited the resolution of the GWA analysis resulting in large genomic segments being significantly associated with each miRNA expression profile. In some cases, this limited our ability to conduct the conditional analysis on a peak SNP due to high correlations between genotypic frequencies of SNPs in close proximity. However, we have shown here the successful utilization of this dataset for identifying local- and distant-acting regulators of miRNA expression despite the extent of linkage disequilibrium in this population. The SNP MARC0093624 (SSC15: 122.22 Mb) was associated with five different miRNAs in our GWA analysis, which may lead to the identification of this marker as a miR-eQTL hotspot. While this genomic region did produce a putative regulatory hotspot in mRNA eQTL analyses (Velez-Irizarry et al., 2019), previous literature has reported eQTL-hotspot analyses being subject to high false-positive rates (Breitling et al., 2008). Thus, further analysis is required to confidently define MARC0093624 as a miR-eQTL hotspot. Additionally, while the recent update to the *Sus scrofa* genome assembly (v11.1; Ensembl) is a vast improvement over the previous reference genome, there still exists a lack of accurate genome assembly data for some genomic regions, making classifying a miR-eQTL as local*-* versus distant*-*acting impossible in some cases. For the majority of cases, however, we were successful in classifying the type of regulatory relationship occurring between genomic regions harboring variants and miRNAs exhibiting variation in expression.

## Conclusion

No previous studies evaluating miRNA-eQTL in pigs have been reported. Our genome-wide analysis of miRNA expression profiles successfully identified genomic regions affecting the expression of 17 unique miRNAs, indicating that miRNA expression in this tissue does have a genetic component. We then examined the potential effects miRNAs experiencing variation could have on the phenotypes measured in this population, demonstrating some key miRNAs colocalizing with growth, carcass composition, and meat quality traits. Some of these miRNAs had previously been identified for their involvement in skeletal muscle and adipose tissues, whereas others represented novel findings. While more research is needed to confirm the roles of miRNA regulation in these traits, our work has contributed to the understanding of regulatory mechanisms underlying complex trait phenotypes in pigs.

## Data Availability Statement

The datasets presented in this study can be found in online repositories. The names of the repository/repositories and accession number(s) can be found below: https://www.ncbi.nlm.nih.gov/bioproject/PRJNA363073.

## Ethics Statement

The animal study was reviewed and approved by the Michigan State University All University Committee on Animal Use and Care AUF # 09/03–114-00.

## Author Contributions

CE, JS, and RB conceived and supervised this research. KD contributed to RNA extraction, performed the microRNA bioinformatic and statistical analysis, composed the article, and created the figures and tables. SC developed code for and conducted the phenotypic QTL analysis. DV-I contributed to RNA extraction, developed the code used for statistical analysis, and provided results of the mRNA eQTL analysis and updated phenotypic QTL analysis. NR performed tissue and phenotypic collection and RNA extraction and oversaw RNA sequencing. All authors contributed to the article and approved the submitted version.

## Conflict of Interest

The authors declare that the research was conducted in the absence of any commercial or financial relationships that could be construed as a potential conflict of interest.

## Footnotes

1 www.mirbase.org

2 https://www.github.com/steibelj/gwaR

3 https://cran.r-project.org

4 www.genecards.org

## Supplementary Material

Supplementary Figure 1 Heritability of miRNA expression. Narrow-sense heritability (*h*^2^) of the 295 miRNA expression profiles was estimated by obtaining the ratio of the additive genetic variance and total phenotypic variance parameters resulting from the GBLUP model. Significance of heritability was assessed using LRTs, and FDR was implemented for multiple test correction. The *x*-axis denotes narrow-sense heritability of the miRNA expression profiles; the *y*-axis denotes the log-adjusted *p*-values of the LRTs. Highlighted in red are the 46 miRNAs exhibiting significantly heritable expression in this dataset (FDR < 0.05).

Supplementary Figure 2 Visualization of correlation between SNPs in miR-eQTL peaks failing conditional analysis. This figure shows the results of correlation analyses between SNPs in the miR-eQTL peak for those miR-eQTL that failed the conditional analyses, repeating the GBLUP-based GWA analysis incorporating the peak SNP for each miR-eQTL as a fixed effect. The significantly associated SNPs comprising the miR-eQTL peak for **(A)** miR-184, **(B)** miR-7135-3p, and **(C)** miR-9810-3p were included in each respective correlation analysis. Each square represents the correlation between a pair of SNPs, identified above and on the diagonal in each plot. The strength of each pair’s correlation is depicted by increasingly saturated color; blue shades represent positive correlations and red shades represent negative correlations between two SNPs.
