# Supplementary figures and images for: Integrated Genome-Wide Analysis of MicroRNA Expression Quantitative Trait Loci in Pig Longissimus Dorsi Muscle
Source: Front Genet. 2021 Mar 30;12:644091. doi: 10.3389/fgene.2021.644091 (PMC8042294; doi:10.3389/fgene.2021.644091)

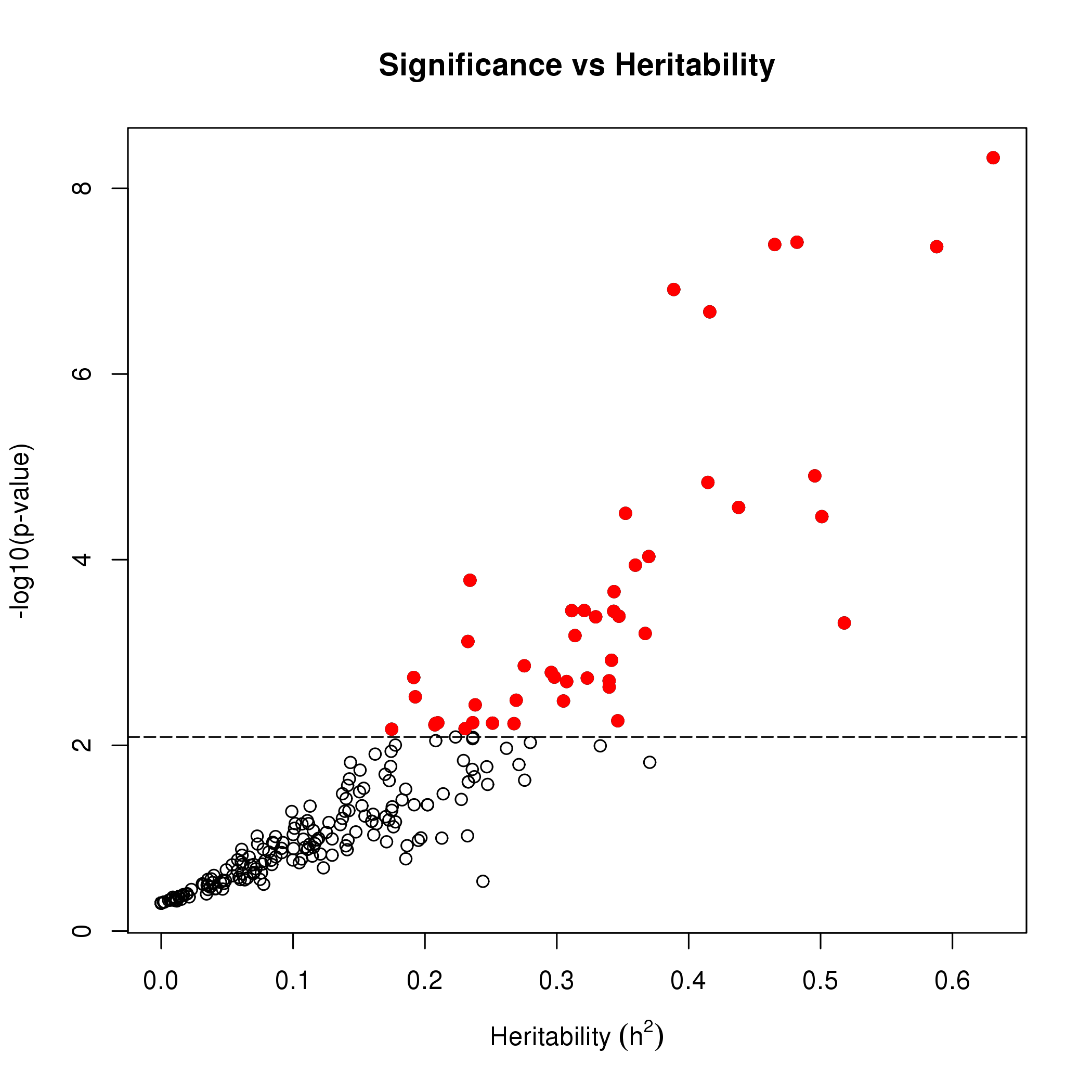

Supplement: Supplementary Figure 1 — Heritability of miRNA expression. Narrow-sense heritability (h2) of the 295 miRNA expression profiles was estimated by obtaining the ratio of the additive genetic variance and total phenotypic variance parameters resulting from the GBLUP model. Significance of heritability was assessed using LRTs, and FDR was implemented for multiple test correction. The x-axis denotes narrow-sense heritability of the miRNA expression profiles; the y-axis denotes the log-adjusted p-values of the LRTs. Highlighted in red are the 46 miRNAs exhibiting significantly heritable expression in this dataset (FDR < 0.05). [file Image_1.TIFF]

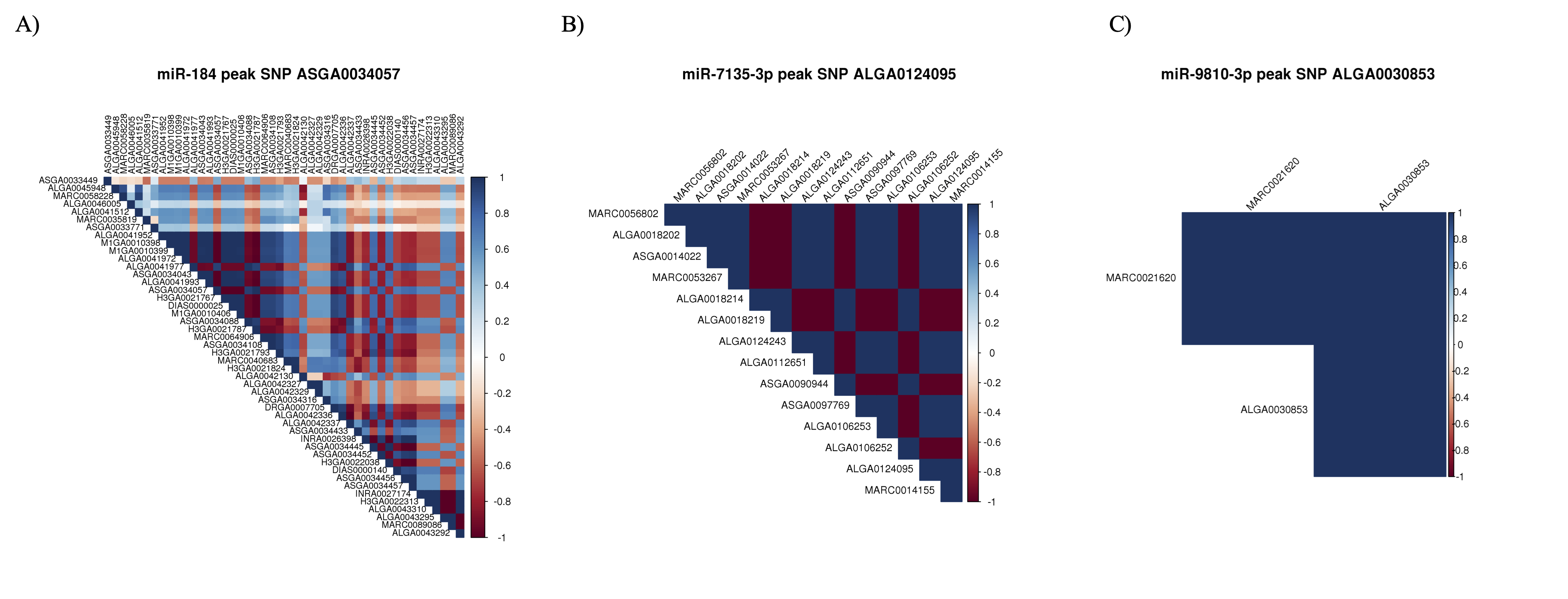

Supplement: Supplementary Figure 2 — Visualization of correlation between SNPs in miR-eQTL peaks failing conditional analysis. This figure shows the results of correlation analyses between SNPs in the miR-eQTL peak for those miR-eQTL that failed the conditional analyses, repeating the GBLUP-based GWA analysis incorporating the peak SNP for each miR-eQTL as a fixed effect. The significantly associated SNPs comprising the miR-eQTL peak for (A) miR-184, (B) miR-7135-3p, and (C) miR-9810-3p were included in each respective correlation analysis. Each square represents the correlation between a pair of SNPs, identified above and on the diagonal in each plot. The strength of each pair’s correlation is depicted by increasingly saturated color; blue shades represent positive correlations and red shades represent negative correlations between two SNPs. [file Image_2.TIFF]
